# Polytobacco product use among current cigarette smokers in Hong Kong, China: results from population surveys (2015–17)

**DOI:** 10.1186/s12889-021-10341-7

**Published:** 2021-02-06

**Authors:** S. L. Chau, M. P. Wang, Y. Wu, D. Y. T. Cheung, A. Kong, V. Lai, T. H. Lam, S. Y. Ho

**Affiliations:** 1 School of Nursing, University of Hong Kong, 21 Sassoon Road, Pokfulam, Hong Kong; 2 Hong Kong Council on Smoking and Health, 183 Queen’s Road East, Wanchai, Hong Kong; 3 School of Public Health, University of Hong Kong, 7 Sassoon Road, Pokfulam, Hong Kong

**Keywords:** Polytobacco product, Current smokers, Smokeless tobacco product, Chinese

## Abstract

**Background:**

Polytobacco product use is increasingly popular, but little is known about the prevalence, trend, and factors of such use particularly in non-western countries.

**Method:**

A representative sample of 1139 current cigarette smokers aged 15+ (84.1% male) were telephone interviewed in Tobacco Control Policy-related Surveys in 2015–2017. Information collected included poly-tobacco use (PTU), smoking and socio-demographic characteristics. Associations of current PTU with related factors were analyzed using logistic regression with adjustment for confounders. Prevalence was weighted by age and sex of current cigarette users in the general population.

**Results:**

Eighty-four point one percent (95% CI 81.4–86.6%) were exclusive cigarette smokers. Fifteen point nine percent (13.4–18.6%) were current polytobacco product users, 12.3% (10.2–14.8%) used one tobacco product and 2.52% (1.59–3.97%) used two tobacco products in addition to cigarette. Cigarette use with cigar was more common (6.28%, 4.75–8.27%), and the least used product with cigarette was e-cigarette (1.05%, 0.44–2.50%). The changes in overall prevalence of PTU by number of products use varied in 3 years. Current PTU was associated with being male (AOR 2.01, 95% CI 1.12–3.61), younger age (AORs range from 1.34–4.65, *P* for trend < .001) and less ready to quit (2.08, 1.09–3.97).

**Conclusions:**

Prevalence of PTU increased slowly by year, one tobacco product use with cigarette was more common. The most used tobacco product with cigarette was cigar. Being male, younger and less ready to quit were associated with current PTU.

**Supplementary Information:**

The online version contains supplementary material available at 10.1186/s12889-021-10341-7.

## Background

Polytobacco product use (PTU) refers to concomitantly using two or more tobacco products, such as current manufactured cigarette (referred as cigarette), cigar, self-rolling cigarette, waterpipe and electronic cigarette (EC) [1]. The worldwide cigarette consumption prevalence was declining in recent decades, but the use of alternative tobacco products was rising globally [2]. In the United States, the sale of cigarette decreased by 18% in 2000–2007, but cigar sales surged by 37% [2]. Concomitant use of EC and waterpipe also increased dramatically from 1.5 to 16% and 4.1 to 7.2% in 2011–2015, respectively [3]. Asian countries reported high prevalence of PTU, especially in Korea [4]. Asian regions accounted for 250 million of polytobacco product users globally [5]. PTU affected more than 70 low, middle and high-income countries [6].

Tobacco industries targeted young population (aged 18–24) and advertised alternative tobacco products as viable smoking cessation and harm reduction tool for quitting cigarette [7]. Youth were more likely to explore different tobacco products [8, 9]. Over 80% of polytobacco product users reported starting PTU in young adult age (aged 18–35) [10] and continued to use in lives [8]. Most research on PTU was conducted in western countries (e.g. U.S.). Several factors were identified associated with PTU, including younger age [2, 11], being male [12], having no intention to quit cigarette [13], and high nicotine dependence [13, 14]. Little is known whether the associations are generalizable to other regions of the world. Particularly in China, one of the biggest tobacco products selling markets in the world.

Foreign and local tobacco companies rapidly expanded their business in China and targeted youth population [15]. Hong Kong is the most westernized city of China with remarkably low cigarette smoking prevalence compared with other Asian regions (10% in 2017) [16], but the use of EC and waterpipe increased in the past few years [17, 18]. In Hong Kong, any sale promotions of tobacco products are prohibited, but marketing and purchasing via social media (e.g. Facebook and Instagram) are not strictly regulated by the government [19]. Many of these tobacco products are readily available for purchase on the Internet without age restriction [20]. Such grey area in regulations created a loophole for promotion and sale of alternative tobacco products for PTU.

We investigated the overall prevalence and trends of PTU. We also identified the association of PTU with socio-demographic characteristics, cigarette dependence, and intention to quit smoking.

## Methods

### Study design

Details of the survey method and the questionnaire used have been reported elsewhere [20]. The Tobacco Control Policy-related Survey (TCPS) was a cross-sectional regular survey commissioned by the Hong Kong Council on Smoking and Health (COSH). The telephone-based survey was conducted by the Public Opinion Program (POP), the University of Hong Kong. This study was an analysis of 2015–17 telephone-based survey, which used two-stage random sampling method. Residential telephone numbers were drawn randomly from residential telephone directories to become seed numbers, another set of numbers were generated using “plus/minus one/two” approach to capture unlisted numbers. One eligible person was selected among the eligible family members using the “next birthday” approach, whose birthday nearest to the survey date was selected at the time of interview. The whole sample of 15,534 Cantonese-speaking respondents (aged 15+) of the 3 surveys included (1) current cigarette smokers, who smoked at least one cigarette in the past 7-day (*N* = 5113); (2) ex-smokers, who had abstained and reported no cigarette smoking in the past 7 days (*N* = 5141) and (3) never smokers (*N* = 5280). The questionnaire comprised core and optional questions. Sociodemographic characteristics, nicotine dependence, and intention to quit were core questions for all current cigarette smokers. Questions related to PTU were optional questions and were administered to randomly selected subsets of a representative sample of current cigarette smokers (*n* = 1139). All respondents in the same subset answered the same sets of optional questions and core questions. All respondents provided oral consent before the telephone interview. Ethical approval was granted by from the Institutional Review Board of the University of Hong Kong/Hospital Authority Hong Kong West Cluster. The study followed STROBE guideline strictly (Appendix 1).

### Measurements

Baseline socio-demographic characteristics were collected, including sex, age, educational attainment, marital status, employment status (economically active and non-active) and monthly household income. Nicotine dependence was measured by Heaviness of Smoking Index (HSI), it comprised of two questions: “How soon after wake up do you smoke your first cigarette? and “How many cigarettes do you smoke per day?” Score 0–1 indicated as light smoker, 2–3 as moderate smoker and 4–6 as heavy smoker [21]. Current cigarette smokers were asked about their intention to quit smoking. It was measured by asking “Do you plan to quit smoking? If yes, what is your planned quit day?” Those reported quit intention within 30-day were defined as ready to quit and beyond 30-day were defined as no intention to quit [22]. Current cigarette smokers were asked if they used other tobacco products (cigars, self-rolling cigarette, waterpipe and electronic cigarette) within 30-day, those responded “Yes” were categorized as current polytobacco product users and “No” as exclusive cigarette smokers. PTU was categorized by number of tobacco products use (one tobacco product and two or more tobacco products use in addition to cigarette).

### Statistical analysis

Inverse probability (of being sampled from the population) weighting based on the sex and age distribution of the Hong Kong adult current cigarette users in 2017 (from census) was conducted to make the sample more representative to Hong Kong population [16]. The associations of socio-demographic characteristics with current PTU were analyzed using multivariable logistic regression. We further examined the associations of HSI and intention to quit with PTU, controlling for sex, age, educational attainment and monthly household income. Results were reported as adjusted odds ratio (AOR). All the analyses were performed using STATA (V. 13.0). Two-tailed *p*-value less than 5% is considered as statistically significant. Listwise deletion was used to handle missing value due to a small proportion of data was missing (< 3%).

## Results

**Fig. 1:**
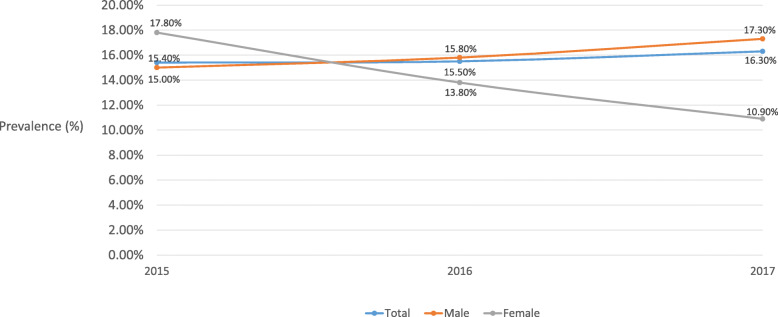
Prevalence of current polytobacco product use by sex, 2015–17 (*n* = 1139)

**Fig. 2:**
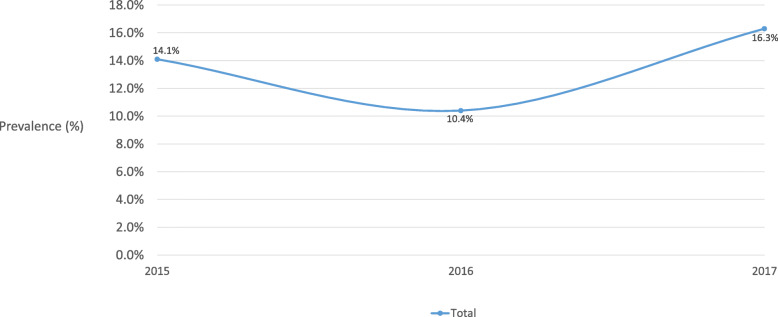
Overall prevalence of one tobacco product use with cigarette, 2015–17 (*n* = 1139)

**Fig. 3:**
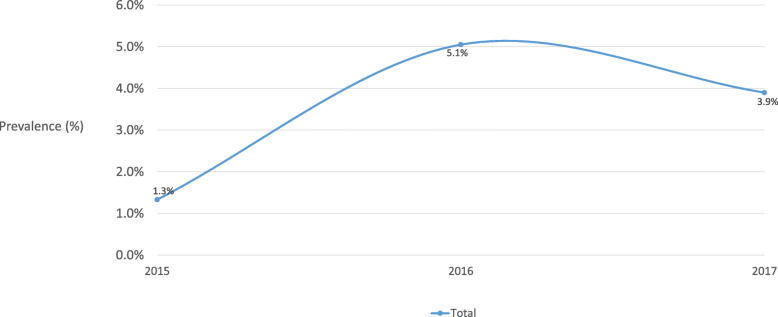
Overall prevalence of two or more tobacco products use with cigarette, 2015–17 (*n* = 1139)

Table 1 shows most of the current smokers were male (84.1%), aged ≥40 (71.2%), had secondary education (59.5%), were economically active (69.6%) and had a monthly household income greater than HK $30,000 (48.0%). 84.1% (95% CI 81.4–86.6%) of current cigarette smokers were exclusive cigarette users. The overall prevalence of current PTU was 15.9% (13.4–18.6%), 12.3% for 2 products (10.2–14.8%), and 2.52% for 3 products (1.59–3.97%). Current PTU increased slightly by year from 15.4–16.3%, 2015–17 (Fig. 1). The overall prevalence of one tobacco product use with cigarette was 6.28% (4.75–8.27%) for cigar, 5.52% (4.15–7.30%) for self-rolling cigarette, 3.77% (2.55–5.56%) for waterpipe, and 1.05% (0.44–2.50%) for EC (Table 2) The trends of overall prevalence of number of tobacco product use with cigarette varied in 3 years (2015–17). For one tobacco product use with cigarette, the prevalence decreased from 14.1% (10.2–19.1%) to 10.4% (6.42–16.5%), then increased to 12.4% (9.53–16.0%) (Fig. 2). For two or more tobacco products use with cigarette, the prevalence increased from 1.33% (0.54–3.25%) to 5.05% (2.35–10.5%), then decreased to 3.90% (2.37–6.34%) (Fig. 3).

**Table 1:** Socio-demographic characteristics of 1139 current cigarette smokers^a^

	Overall sample *n* = 1139 n (%)	Exclusive cigarette smoker *n* = 987 (84.2%, 81.4–86.6%) n (%)
Sex		
Male	932 (84.1)	805 (83.7)
Female	207 (15.9)	182 (16.3)
Age		
15–29	88 (10.4)	60 (8.60)
30–39	107 (18.4)	80 (16.0)
40–49	166 (24.6)	143 (25.3)
50–59	250 (22.2)	221 (23.3)
≥ 60	443 (24.4)	408 (26.8)
Educational attainment ^b^		
Primary or below	241 (15.1)	223 (16.3)
Secondary	660 (59.5)	580 (61.7)
Tertiary or above	231 (25.4)	178 (22.0)
Marital status		
Single	204 (24.5)	155 (21.5)
Married/cohabited	785 (64.9)	699 (67.6)
Divorced/widowed	143 (10.6)	127 (10.9)
Employment		
Economically active	641 (69.6)	540 (68.5)
Economically non-active	492 (30.4)	441 (31.5)
Monthly income ^c^		
≥ 30,000	407 (48.0)	337 (46.4)
0000–29,999	171 (18.5)	148 (18.3)
≤ 19,999	397 (33.5)	356 (35.3)

^a^ The proportions were weighted by age and sex distribution of adult current cigarette users in Hong Kong 2017, the observations (n) were unweighted

^b^ Proportion add up is not 100% due to rounding

^c^ US$ 1 = HK$ 7.8

**Table 2:** Overall prevalence of one tobacco product use with cigarette of 1139 current cigarette smokers

Tobacco products	n	% ^a^ (95% CI)
Cigar + cigarette	57	6.28 (4.75–8.27)
Self-rolling cigarette + cigarette	57	5.52 (4.15–7.30)
Waterpipe + cigarette	33	3.77 (2.55–5.56)
Electronic cigarette + cigarette	7	1.05 (0.44–2.50)

^a^Prevalence was weighted by age and sex distribution of adult current cigarette users in Hong Kong 2017, the observations (n) were unweighted

For socio-demographic characteristics variables, being male was associated with current PTU (AOR 2.01, 95% CI 1.12–3.61). Inverse association was found between age and current PTU; the AOR increased with younger age and the association remains strongly significant after adjustment. (AORs range from 1.34–4.65, P for trend less than 0.001) (Table 3). No intention to quit cigarette smoking was associated with current PTU (AOR 2.08, 95% CI 1.09–3.97). HSI was not associated with current PTU before and after adjustment (Table 4).

**Table 3:** Associations of socio-demographic characteristics with current polytobacco product use in 1139 current smokers

	Current polytobacco product use		
	n (%)	Crude OR (95% CI)	AOR (95% CI) ^a^ (*n* = 905)
Sex			
Female	207 (18.2)	1	1
Male	932 (81.8)	1.15 (0.73–1.82)	2.01 (1.12–3.61)*
Age (years)			
≥ 60	443 (42.0)	1	1
50–59	250 (23.7)	1.53 (0.91–2.57)	1.34 (0.69–2.58)
40–49	166 (15.8)	1.87 (1.07–3.28) *	1.95 (0.94–4.06)
30–39	107 (10.2)	3.93 (2.26–6.86) ***	4.15 (1.90–9.07) ***
15–29	88 (8.30)	5.44 (3.09–9.58) ***	4.65 (1.87–11.6) ***
P for trend		< 0.001	< 0.001
Educational attainment			
Primary or below	241 (21.3)	1	1
Secondary	660 (58.3)	1.71 (1.01–2.92) *	1.10 (0.59–2.05)
Tertiary or above	231 (20.4)	3.69 (2.09–6.52) ***	1.32 (0.64–2.72)
P for trend		< 0.001	0.30
Marriage			
Married/cohabited	785 (69.4)	1	1
Divorced/widowed	143 (12.6)	1.02 (0.58–1.80)	1.18 (0.62–2.24)
Single	204 (18.0)	2.57 (1.74–3.80) ***	1.06 (0.59–1.89)
Employment			
Economically non-active	492 (43.4)	1	1
Economically active	641 (56.6)	1.62 (1.13–2.32) **	0.82 (0.47–1.44)
Monthly household income (HK$) ^b^			
≤ 19,999	397 (40.7)	1	1
20,000–29,999	171 (17.5)	1.35 (0.78–2.33)	0.87 (0.46–1.65)
≥ 30,000	407 (41.7)	1.80 (1.19–2.73) **	1.30 (0.78–2.16)
P for trend		< 0.01	0.21

^a^ All variables were mutually adjusted

^b^ US$ 1 = HK$ 7.8

* *P* < 0.05; ** *P* < 0.01; *** *P* < 0

**Table 4:** Associations of cigarette dependence and intention to quit with current polytobacco product use in 1139 current smokers

	Current polytobacco product use		
	n (%)	Crude OR (95% CI)	AOR (95% CI) ^a^ (*n* = 902)
Heaviness of Smoking Index			
0–2 (light)	538 (54.4)	1	1
3–4 (medium)	384 (38.8)	1.16 (0.79–1.69)	1.35 (0.87–2.08)
5–6 (high)	67 (6.77)	0.94 (0.43–2.05)	0.90 (0.36–2.24)
Intention to quit			
Within 30 days	170 (15.1)	1	1
No intention to quit	957 (84.9)	1.73 (0.99–3.02)	2.08 (1.09–3.97)*

^a^ Adjusting for sex, age, educational attainment and income

* *P* < 0.05; ** *P* < 0.01; *** *P* < 0.001

## Discussion

We identified the prevalence, trends, and factors associated with PTU. We found the prevalence of PTU was slowly increasing in 3 years, one tobacco product use with cigarette was more common, cigar and cigarette use has the highest prevalence compared with other patterns of PTU. The trend changes in overall prevalence of number of tobacco product use with cigarette varied in 3 years. Being male and less ready to quit were associated with current PTU. We also found strong association of being younger age with current PTU.

This is the first large scale study to examine the prevalence, trends, and factors associated with PTU from a large representative sample of Chinese general population. We observed that the prevalence of current PTU (15.9%) was lower than the U.S. (38.7%) [23], but was higher than some large European countries, such as Ukraine (12.0%) and Turkey (12.3%) [24]. Studies from other countries (e.g. U.S., Canada and Egypt) also showed consistent findings of increasing prevalence of PTU [24, 25]. The slow increasing PTU may be driven by relatively strict regulation of cigarettes sales compared with alternative tobacco products. Small geographical area (e.g. Hong Kong) along with high penetration of smartphone also contributes to increasing PTU via frequent social media exposure of alternative tobacco products [26]. Estimates indicated using one tobacco product was more common than using two tobacco products in addition to cigarette (12.3% vs. 2.52%). Among this, cigar was the most used tobacco product (6.28%) compared with EC (1.05%). This suggested EC was less popular for PTU. Dual use of cigar and cigarette was associated with less negative perception toward cigarette use and quit attempts [27], tobacco intervention program specific to this subgroup of smokers is warranted to prevent the growth of this type of PTU. The trend changes in overall prevalence of number of tobacco product use with cigarette varied in 3 years, this suggested these smoking behaviors had not been routinized. PTU is in the early stage of epidemic in Hong Kong, continuous monitoring and early intervention are warranted to prevent progression of long-term PTU.

Although studies on social norm and risk perception of PTU are limited, some studies had examined the socio-demographic characteristics associated with PTU [11, 28, 29]. Consistent with previous studies in other countries [2, 9, 30, 31], our findings showed that being male and younger age were positively associated with current PTU. Men were reported with higher risk-taking and sensation-seeking behaviors, such as drug or alcohol abuse and smoking experimentation [32, 33]. Dose-response relationship was observed between age and current PTU; being younger was significantly more likely to use multiple tobacco products in addition to cigarette. The association remains significant and robust after accounting for confounders. Tobacco industries mainly target young population by introducing these products (e.g. waterpipe) in different flavors and promoting the products as less harmful than cigarette on social media [34]. This age group of current smokers perceived PTU as less harmful, less addictive and more fashionable than smoking cigarettes [35, 36]. Many of cigar and waterpipe lounges were also densely located in nightlife district, where the modern and luxurious environments attracted large amount of young smokers [18]. Our findings highlighted that younger age was a significant factor for current PTU, further studies are needed to explore their knowledge and perception about PTU.

We extend the understanding of smoking and quitting behaviors with current PTU. Current smokers with no intention to quit cigarette had higher odds of PTU. Other studies found positive association between higher quit attempts and PTU [11, 37]. The results were explained by the finding that polytobacco product users had lower intention to quit smoking cigarette because they experienced less cessation success despite with higher quit attempts [22]. Most of the polytobacco product users were young, young smokers were associated with lower intention to quit smoking cigarette compared with other age groups [12, 38]. Instead of complete abstinence from smoking cigarettes, young smokers switched from exclusive cigarette users to polytobacco product users for smoking experimentation [11, 39, 40]. Some studies showed positive association between nicotine dependence and PTU [8, 12–14], this was inconsistent with our finding. Heaviness of Smoking Index only measured the degree of nicotine dependence from smoking cigarette, it is possible that current smokers who concomitantly used cigarette with other tobacco products did not solely rely on cigarette to acquire nicotine and therefore smoked less compared with exclusive cigarette smokers. Such behavior is a barrier for smoking cessation treatment, as this specific group of smokers rarely seek cessation services and make them harder to quit [37]. Future longitudinal studies are needed to specifically follow polytobacco product users to investigate their smoking behaviors and cessation outcomes.

This study has some limitations. As the analysis was based on repeated cross-sectional data, a longitudinal study is needed to further explore the detailed trend changes of PTU and the temporal relations of smoking and quitting behaviors with PTU. As smoking is generally considered unacceptable in Chinese culture, respondents may subject to social desirability bias due to nature of the survey. Socially desirable responses were likely to occur in response to sensitive questions, such as number of cigarettes smoked and tobacco products used [41, 42], therefore the prevalence may be underestimated. Confidentiality was assured before the interview began, but data collected from the lane line surveys were self-reported and answers might still subject to measurement and reporting bias due to underreporting, attenuation of associations were possible [43].

## Conclusions

We found the prevalence of PTU increased slowly by year. One tobacco product use with cigarette was more common and the most used tobacco product with cigarette was cigar. Being male, younger and less ready to quit were associated with current PTU. These factors can be used as the important indicators to identify specific vulnerable groups for controlling the rise of PTU in future.

## Supplementary Information

**Additional file 1 **STROBE Statement, Checklist of items that should be included in reports of *cross-sectional studies*.

## Data Availability

The datasets used and/or analysed during the current study are available from the corresponding author on reasonable request.

## Abbreviations

EC: Electronic cigarette
PTU: Poly-tobacco product use
TCPS: Tobacco Control Policy-related Survey
COSH: The Hong Kong Council on Smoking and Health
POP: The Public Opinion Program
HSI: Heaviness of Smoking Index
AOR: Adjusted odds ratio
