# Supplementary material for: Polytobacco product use among current cigarette smokers in Hong Kong, China: results from population surveys (2015–17)
Source: BMC Public Health. 2021 Feb 6;21:307. doi: 10.1186/s12889-021-10341-7 (PMC7866759; doi:10.1186/s12889-021-10341-7)
Supplement: Supplementary file 1 — Additional file 1 STROBE Statement, Checklist of items that should be included in reports of cross-sectional studies. [file 12889_2021_10341_MOESM1_ESM.docx]

**Appendix 1.**

STROBE Statement—Checklist of items that should be included in reports of ***cross-sectional studies***

|  | Item No | Recommendation |  |
| --- | --- | --- | --- |
| **Title and abstract** | 1 | (*a*) Indicate the study’s design with a commonly used term in the title or the abstract | Abstract, p.1, line 17 |
|  |  | (*b*) Provide in the abstract an informative and balanced summary of what was done and what was found | Abstract, p.1, line 16 |
| Introduction | | |  |
| Background/rationale | 2 | Explain the scientific background and rationale for the investigation being reported | Background section, p.2, line 7 |
| Objectives | 3 | State specific objectives, including any prespecified hypotheses | Background section, p.3, line 12-15 |
| Methods | | |  |
| Study design | 4 | Present key elements of study design early in the paper | Methods section, p. 3, line 19 |
| Setting | 5 | Describe the setting, locations, and relevant dates, including periods of recruitment, exposure, follow-up, and data collection | Methods section, p. 3, line 19 |
| Participants | 6 | (*a*) Give the eligibility criteria, and the sources and methods of selection of participants | Methods section, p.4, line 1 |
| Variables | 7 | Clearly define all outcomes, exposures, predictors, potential confounders, and effect modifiers. Give diagnostic criteria, if applicable | Methods sections, p.5, line 1 |
| Data sources/ measurement | 8 | For each variable of interest, give sources of data and details of methods of assessment (measurement). Describe comparability of assessment methods if there is more than one group | Methods section, p.5, line 3 |
| Bias | 9 | Describe any efforts to address potential sources of bias | Nil |
| Study size | 10 | Explain how the study size was arrived at | Methods section, p.4, line 10 |
| Quantitative variables | 11 | Explain how quantitative variables were handled in the analyses. If applicable, describe which groupings were chosen and why | Methods section, p.5, line 3 |
| Statistical methods | 12 | (*a*) Describe all statistical methods, including those used to control for confounding | Methods section, p.5. line 17 |
|  |  | (*b*) Describe any methods used to examine subgroups and interactions | Nil |
|  |  | (*c*) Explain how missing data were addressed | Methods section, p.6, line 5 |
|  |  | (*d*) If applicable, describe analytical methods taking account of sampling strategy | Nil |
|  |  | (*e*) Describe any sensitivity analyses | Methods section, p.6, line 5 |
| Results | | |  |
| Participants | 13 | (a) Report numbers of individuals at each stage of study—eg numbers potentially eligible, examined for eligibility, confirmed eligible, included in the study, completing follow-up, and analysed | Results section, p.6, line 7 |
|  |  | (b) Give reasons for non-participation at each stage | Nil |
|  |  | (c) Consider use of a flow diagram | Nil |
| Descriptive data | 14 | (a) Give characteristics of study participants (eg demographic, clinical, social) and information on exposures and potential confounders | Results section, p.6. line 7-16 |
|  |  | (b) Indicate number of participants with missing data for each variable of interest | Methods section, p.6, line 5 |
| Outcome data | 15 | Report numbers of outcome events or summary measures | Results section, p.7, line 1 |
| Main results | 16 | (*a*) Give unadjusted estimates and, if applicable, confounder-adjusted estimates and their precision (eg, 95% confidence interval). Make clear which confounders were adjusted for and why they were included | Results section, p. 6, line 7-16 |
|  |  | (*b*) Report category boundaries when continuous variables were categorized | Methods section, p.5, line 4 |
|  |  | (*c*) If relevant, consider translating estimates of relative risk into absolute risk for a meaningful time period | Nil |
| Other analyses | 17 | Report other analyses done—eg analyses of subgroups and interactions, and sensitivity analyses | Results section, p. 7, line 9 |
| Discussion | | |  |
| Key results | 18 | Summarise key results with reference to study objectives | Discussion section, p.7. line 17-22 |
| Limitations | 19 | Discuss limitations of the study, taking into account sources of potential bias or imprecision. Discuss both direction and magnitude of any potential bias | Discussion section, p.10, line 13-22 |
| Interpretation | 20 | Give a cautious overall interpretation of results considering objectives, limitations, multiplicity of analyses, results from similar studies, and other relevant evidence | Discussion section, p.8, line 1 |
| Generalisability | 21 | Discuss the generalisability (external validity) of the study results | Discussion section, p.8, line 4 |
| Other information | | |  |
| Funding | 22 | Give the source of funding and the role of the funders for the present study and, if applicable, for the original study on which the present article is based | Funding section, p.12, line 19-21 |
